# Bovine Peripheral Blood-Derived Mesenchymal Stem Cells (PB-MSCs) and Spermatogonial Stem Cells (SSCs) Display Contrasting Expression Patterns of Pluripotency and Germ Cell Markers under the Effect of Sertoli Cell Conditioned Medium

**DOI:** 10.3390/ani14050803

**Published:** 2024-03-05

**Authors:** Moisés N. Segunda, Carlos Díaz, Cristian G. Torres, Víctor H. Parraguez, Mónica De los Reyes, Oscar A. Peralta

**Affiliations:** 1 Faculty of Veterinary and Animal Sciences, University of Chile, Santiago 8820808, Chile; segundamois@gmail.com (M.N.S.); crtorres@uchile.cl (C.G.T.); vparragu@uchile.cl (V.H.P.); mdlreyes@uchile.cl (M.D.l.R.); 2 Doctorate Program of Forestry, Agriculture, and Veterinary Sciences (DCSAV), University of Chile, Santiago 8820808, Chile; 3 Faculdade de Medicina Veterinária, Universidade José Eduardo dos Santos, Bairro Santo António-Avenida Nuno Alvarez, Huambo 555, Angola; 4 Doctorate Program in Sciences, UNED, Bravo Murillo 38, 28015 Madrid, Spain; c.diaz.boudon@gmail.com; 5 Escuela de Medicina Veterinaria, Facultad de Agronomía e Ingeniería Forestal, Facultad de Ciencias Biológicas y Facultad de Medicina, Pontificia Universidad Católica de Chile, Vicuña Mackenna 4860, Macul, Santiago 7820436, Chile

**Keywords:** mesenchymal stem cells, spermatogonial stem cells, Sertoli cells, germ cell differentiation, conditioned medium

## Abstract

**Simple Summary:**

The development of a biological system for gamete production may be a ground-breaking strategy for the treatment of infertility in humans, improvement of genetic traits in livestock, and conservation of endangered animal species. In testis, sperm derives from spermatogonial stem cells (SSCs) in a microenvironment controlled by surrounding Sertoli cells (SCs). Mesenchymal stem cells (MSCs) derived from peripheral blood (PB-MSCs) are a novel source of multipotent progenitors that can potentially differentiate under in vitro conditions into germ cells (GCs) and sperm. The objective of the present study was to compare the potential for in vitro GC differentiation of bovine PB-MSCs and SSCs exposed to culture medium derived from Sertoli cells (SCs/CM) over a period of 21 days. Samples were collected every 7 days for 21 days and analyzed for expression of pluripotency (*OCT4*, *NANOG*, and *SOX2*), GC (*DAZL* and *PIWIL2*), and MSC (*CD73* and *CD105*) markers using quantitative PCR and immunofluorescence. SSCs cultured with SCs/CM increased the expression of *NANOG*, *PIWIL2*, and *DAZL*, while PB-MSCs cultured under the same condition only increased the expression of OCT4 and *DAZL*. In conclusion, these results suggest that PB-MSCs and SSCs activate distinctive and contrasting expression patterns after exposure to SCs/CM and during differentiation into GCs.

**Abstract:**

In vitro gamete derivation has been proposed as an interesting strategy for treatment of infertility, improvement of genetic traits, and conservation of endangered animals. Spermatogonial stem cells (SSCs) are primary candidates for in vitro gamete derivation; however, recently, mesenchymal stem cells (MSCs) have also been proposed as candidates for germ cell (GCs) differentiation mainly due to their transdifferentiating capacity. The objective of the present study was to compare the potential for GC differentiation of bovine peripheral blood-derived MSCs (PB-MSCs) and SSCs under the effect of conditioned medium (CM) derived from Sertoli cells (SCs/CM). Samples were collected every 7 days for 21 days and analyzed for pluripotent, GC, and MSC marker expression. The absence of OCT4 and the increased (*p* < 0.05) expression of *NANOG* seems to play a role in SSC differentiation, whereas the absence of *NANOG* and the increased expression (*p* < 0.05) of OCT4 may be required for PB-MSC differentiation into GCs. SSCs cultured with SCs/CM increased (*p* < 0.05) the expression of *PIWIL2* and *DAZL*, while PB-MSCs cultured under the same condition only increased (*p* < 0.05) the expression of *DAZL*. Overall, the patterns of markers expression suggest that PB-MSCs and SSCs activate different signaling pathways after exposure to SCs/CM and during differentiation into GCs.

## 1. Introduction

MSCs are a novel source of multipotent progenitors that can differentiate into mesodermal lineages including osteocytes, chondrocytes, and adipocytes through the process of transdifferentiation [1]. It has also been reported that MSCs are able to differentiate under in vitro conditions towards endodermal (hepatocyte) and ectodermal (neuronal) lineages [2,3,4]. These studies indicate that the differentiation potential of MSCs may be greater than initially reported. MSCs are widely distributed and can be isolated from various tissue sources including adipose tissue (AT), bone marrow (BM), umbilical cord (UC) [5], and also peripheral blood (PB) [6,7]. PB is an interesting alternative source of MSCs, since its collection is simple and requires minimal invasion compared to BM and AT, whose acquisition requires invasive and painful procedures. In addition, PB may be obtained from readily available blood banks, and its collection lacks ethical concerns compared to fetal tissues [8]. Despite these advantages, it has been reported that PB contains a low percentage of MSCs at a steady state, a condition that may affect the isolation process [8,9].

Given their multipotent property, the potential of MSCs for differentiation into GCs has been explored to develop a system for gamete production [10,11,12,13]. This technology has emerged as a potential strategy for the treatment of infertility in humans, for genetic improvement in livestock, or for conservation of endangered animal species. Through different protocols, it has been shown that MSCs can acquire an early phenotype of GCs under in vitro conditions (10). This phenotype may be achieved after exposure to factors involved in GC differentiation and spermatogenesis including retinoic acid (RA), bone morphogenetic protein 4 (BMP4), or transforming growth factor β1 (TGF β1) [10,14]. The ability of MSCs to reach this initial stage of GCs has also been explored through the overexpression of GCs genes that control differentiation including *DAZL, STRA8*, and *BOULE* [11]. An additional in vitro differentiation approach comprises the co-culture of MSCs with SCs [12,13], or the exposure of MSCs to conditioned media (CM) collected from SC cultures (SCs/CM) [15,16,17]. Several factors contained in the CM, including RA, BMP4, and TGF β1, are responsible for the control of spermatogenesis in vivo and may induce GC differentiation under in vitro conditions. Treatment of human UC-MSC with SCs/CM supplemented with RA induced down-regulation of pluripotent (OCT4 and NANOG) and upregulation of GC (STRA8 and PRM1) markers [16]. Moreover, rat BM-MSCs treated with SCs/CM were able to survive and were located at the basement membranes of the recipient seminiferous tubules [17]. Overall, these studies have brought promising results, which suggests that MSCs may be used as a candidates for in vitro derivation of GCs.

SSCs constitutes the precursor cell line of spermatogenesis and sperm production in vivo [18]. During spermatogenesis, SSCs are surrounded by SCs, which give them a microenvironment or cellular niche that delivers factors that control self-renewal, proliferation, and differentiation [18]. Among these factors, RA, BMP4, and TGF-β1 are involved in the induction of cell proliferation and differentiation through the expression of germline-specific genes and the restauration the germline competence [15,18]. In this function, SCs regulate the composition of the fluid in the seminiferous tubule, which is rich in androgens, estrogens, and other essential factors for proliferation and differentiation of GCs [19,20,21,22]. Thus, SCs are essential players in the testicular niche, where they represent the only somatic cells that have close connection with GCs and modulate their cell cycle [23,24,25,26].

In general, CM includes proteins detached from the cell surface and intracellular proteins released through the non-classical secretion pathway or exosomes [27]. In the case of SCs/CM, this includes numerous enzymes, growth factors, cytokines and hormones that provides a micro-environment for GCs, at all stages of development. In previous studies, SCs/CM has been used to induce differentiation of BM-MSCs into GCs, through the evaluation of the expression of the GC-specific marker Mvh [23]. A similar experimental approach could be applied to PB-MSCs, and SSCs may serve as comparative positive controls to assess the differentiation process into GCs. The objective of the present study was to compare the GC differentiation potentials of bovine PB-MSCs and SSCs in conventional 2D culture systems under the inducing effect of the SCs/CM.

## 2. Materials and Methods

### 2.1. Ethics

All experimental procedures were previously approved by the Institutional Committee for the Care and Use of Animals (CICUA) of the University of Chile (certificate 19266-VET-UCH).

### 2.2. Experimental Design

PB-MSCs were collected and polled from three Angus beef bulls (age 1–3 years) belonging to the Faculty of Veterinary and Animal Sciences from the University of Chile. Each pool represented a biological replicate, and experiments and analyses were performed thrice. Pooling tissue samples was performed to reduce individual biological variation. SCs and SSCs were isolated from adult bull testis (*n* = 10) derived from a local abattoir. All cell types were characterized according to the gene expression of specific markers for MSCs (*+CD73, +CD105, +CD90, -CD34*, and *-CD45*), SSCs (+*UCHL1*, *PLZF*, and +*CD90*) and SCs (+*WT1* and +*AR*) using quantitative PCR (Q-PCR). Cells were also characterized for the protein expression of markers for MSCs (*Cd73*), SSCs (*Uchl1*), and SCs (*Wt1*) using immunofluorescence. PB-MSCs and SSCs (2.5 × 10^4^ cells/cm^2^) were cultured separately with SCs/CM in 2D culture systems. Experimental controls corresponded to PB-MSCs, SSCs, and SCs (2.5 × 10^4^ cells/cm^2^) cultured separately with conventional media. Samples from each culture were collected on days 0, 7, 14, and 21. Differentiation into GCs of PB-MSCs and SSCs was determined by quantifying the gene expression of the pluripotency markers *OCT4*, *NANOG*, and *SOX2*; GCs markers *FRAGILIS*, *STELLA*, *DAZL*, *PIWIL2*, *VASA, SCP3*, and *STRA8* by Q-PCR; and *Oct4*, *Nanog*, *Dazl*, and *Piwil2* by immunofluorescence.

### 2.3. Isolation, Purification, and Characterization of Bull PB-MSCs

**Figure 1:**
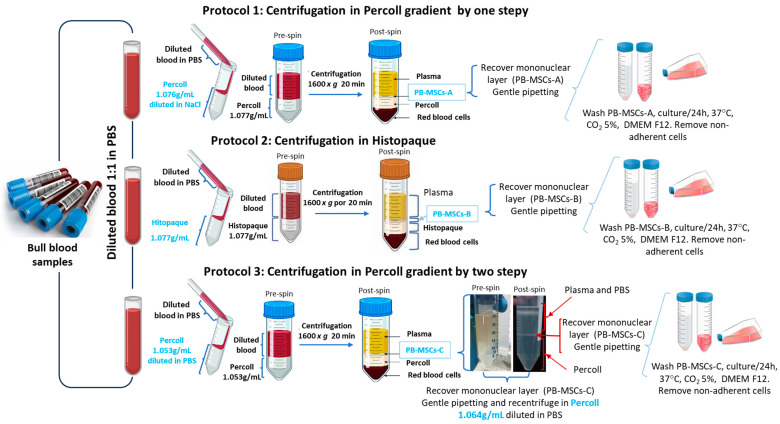
Schematic representation of bovine PB-MSCs isolation and purification by three protocols. Protocol 1. PB MSCs-A were isolated by centrifugation of blood sample in Percoll gradient 1.076 g/mL diluted in NaCl. Protocol 2. PB MSCs-B were isolated by centrifugation of blood sample in Histopaque gradient 1.076 g/mL diluted in NaCl. Protocol 3. PB-MSCs-A were isolated by two-step centrifugation of blood sample in Percoll gradient 1.053 g/mL and 1.064 g/mL diluted in PBS. For each protocol, PB-MSCs were isolated by their adhesion to plastic culture plates.

PB-MSCs were isolated according to three previously reported protocols [7,28,29] (Figure 1). Briefly, blood samples (25 mL per animal) were collected from the coccygeal vein of 1- to 3-year-old bulls using vacutainer tubes containing heparin. Blood samples were kept at 4 °C and transported to the laboratory within 1 h after collection. For all protocols, blood samples were diluted 1:1 in phosphate-buffered saline (PBS; pH: 7.4). Three protocols for isolation of PB-MSCs were evaluated [30]. Protocol 1: PBS-diluted blood (1:1) was poured onto 20 mL of Percoll (1.076 g/mL; Biochrom AG, Berlin, Germany) diluted with 1.5 M NaCl and was centrifugated 1600× *g* for 20 min. The mononuclear cell interface (PB-MSCs-A) was recovered, subsequently washed in PBS, and resuspended in PB-MSC culture medium containing low-glucose DME-F12 (Dulbecco’s Modified Eagle F12) medium supplemented with 10% fetal bovine serum (FBS), 100 µg/mL of amphotericin B, 100 µg/mL of streptomycin, and 100 IU/mL of penicillin (Gibco, Life Technologies, Carlsbad, CA, USA). Protocol 2: PBS-diluted blood (1:1) was centrifugated 1600× *g* for 20 min in gradient of 20 mL Histopaque 1.077 g/mL (Sigma-Aldrich, St Louis, MI, USA). The monoclonal cell interface (PB-MSCs-B) was collected, washed in PBS, and resuspended in PB-MSC culture medium. Protocol 3: Blood diluted in PBS (1:1) was centrifuged at 1600× *g* for 20 min in two consecutive moments, first in 20 mL Percoll at 1.053 g/mL diluted with PBS, and then the average interface of monoclonal cells (PB-MSCs-C) was collected and recentrifuged in 20 mL Percoll at 1.064 g/mL, also diluted with PBS. Then the interface of monoclonal cells (PB-MSCs-C) was collected washed in PBS, and resuspended in PB-MSC culture medium. Subsequently, cells obtained from each protocol were cultured separately in T25 culture bottles (Corning, Toronto, ON, Canada) in PB-MSC culture medium and incubated in an atmosphere of humidified air with 5% CO_2_ and 38 °C. Non-adherent cells were discarded after 48 h by changing the culture medium. Isolated cell colonies were maintained in PB-MSC culture medium until reaching ~75% confluency. Cells were then detached with 0.5% trypsin-EDTA (Invitrogen, BRL, Burlington, ON, Canada) for subculture and used in subsequent experiments. The morphological features of PB-MSCs were examined and photographed using phase contrast microscopy. The expression of cell surface markers, both mesenchymal (+*CD73*, +*CD90*, and +*CD105*) and hematopoietic (-*CD34* and -*CD45*) were evaluated in PB-MSCs-A, PB-MSCs-B, and PB-MSCs-C by Q-PCR. For this analysis, SCs, SSCs-A, and SSCs-B were used as controls. *CD73* protein expression was evaluated by immunofluorescence in PB-MSCs-B to characterize the cell phenotype established by the International Society for Cell Therapy (ISCT) [1]. For this analysis, SCs and SSCs-A were used as controls.

### 2.4. Isolation, Purification, and Characterization of Bull SCs

SCs were isolated from adult bull testicular tissue by sequential enzymatic digestion according to a previously published protocol [12,13]. Testicular tissue was mechanically dissociated using scissors and forceps. Subsequently, an enzymatic digestion of the tissue was performed in DME-F12 medium with L-glutamine supplemented with 2 mg/mL of collagenase type I and 2 mg/mL of DNase I (Sigma-Aldrich) for 45 min. The digestion reaction was blocked by adding SCs culture medium containing DME-F12 supplemented with 10% FBS, 100 IU/mL penicillin, 100 µg/mL streptomycin, and 100 µg/mL of amphotericin B. Then, the cell suspension was centrifuged at 500× *g* for 10 min at 4 °C and the pellet was filtered through a 100 µm mesh (Corning). Cells were aliquoted and seeded in T25 bottles with SCs culture medium. Cultures of SCs were subsequently incubated at 38 °C in a humidified atmosphere with 5% CO_2_. After 4 h, the supernatant containing GCs was removed, and cells adherent to the plastic were washed twice with PBS and subsequently cultured in fresh medium. To obtain cultures containing 80–90% of SCs, cultures were hypotonically treated after 48 h, with 20 mM Tris-HCl (pH 7.4) for 2.5 min to lyse residual GCs. Subsequently, plates were washed twice with PBS, and after changing the medium, cells were cultured in an atmosphere with 5% CO_2_ at 38 °C. When reaching 80% to 90% confluency, adherent cells were detached using 0.5% trypsin-EDTA and transferred to new plates for expansion and removal of residual GCs. During cultivation, non-adherent GCs were removed by repeated washing. The morphological features of SCs were examined and photographed using phase contrast microscopy. A testicular extract (TE) sample was used as a positive control for Q-PCR analyses; this sample was prepared by mechanical dissociation of testicular tissue pieces in lysis buffer (Zymo Research, Irvine, CA, USA). The expression of SCs markers *WT1* and *AR* was evaluated in SCs by Q-PCR. For this analysis, PB-MSCs-A, PB-MSCs-B, PB-MSCs-C, SSCs-A, SSCs-B, and TE were used as controls. The protein expression of *Wt1* was evaluated by immunofluorescence in SCs. For this analysis, PB-MSCs-B and SSC-A were used as controls.

### 2.5. Isolation, Purification, and Characterization of Bull SSCs

SSCs were isolated from adult testicular tissue by a modified adhesion separation protocol previously published [31]. After testis decapsulation, testicular tissue was mechanically dissociated by a two-step mechanical and enzymatic digestion. First, the tissue was sectioned into small pieces and incubated in the enzyme solution that included DME-F12 supplemented with 0.5 mg/mL collagenase type I, 0.5 mg/mL trypsin-EDTA (Invitrogen), and 0.5 mg/mL DNase I for approximately 45 min with shaking and pipetting at 38 °C. The digested testicular tissue was washed in DMEM, and after precipitation, the supernatant containing interstitial cells from the seminiferous tubules was discarded. The remaining tissue was digested during the second stage of enzymatic digestion until separation of its constituent cells. The cell suspension was two-step filtrated through 100 µm and 40 µm filters, and then the cell suspension was cultured twice for 24 h in SCs culture medium. Separation of SSCs (non-adherent cells) from SCs (adherent cells) was performed by differential plate method during 24 h of incubation. For this, testicular cells were transferred to new culture bottles and maintained overnight in SCs culture medium at 38 °C in 5% CO_2_. After this step, floating cells were collected (SSCs-A) and separated from adherent cells (SSCs-B). Both cell types (SSCs-A and SSCs-B) were centrifuged separately at 1800× *g* for 5 min and were characterized by expression of specific biomarkers including *UCHL1, PLZF*, and *CD90* [31] using Q-PCR. For this analysis, PB-MSCs-A, PB-MSCs-B, PB-MSCs-C, SCs, and TE were used as controls. Additionally, the protein expression of *Uchl1* was evaluated in SSCs-A by immunofluorescence. For this analysis, PB-MSCs-B and SCs were used as controls.

### 2.6. Culture of PB-MSCs and SSCs under SCs/CM Conditions

SCs/CM was collected from primary SCs after two days of culture and then centrifuged at 1800× *g* for 5 min to remove residual cells and reconstituted with 50% DME/F-12 supplemented with 10% fetal bovine serum (FBS), 100 μg/mL amphotericin B, 100 μg/mL streptomycin, and 100 IU/mL penicillin. Subsequently, PB-MSCs or SSCs were cultured in adherent monolayers with SCs/CM for 21 days at 38 °C in 5% CO_2_. The SCs/CM was changed every two days and then cell samples were collected every 7 days. The GC differentiation capacity of PB-MSCs cultured with SCs/CM (PB-MSCs-SCs/CM) and SSCs cultured with SCs/CM (SSCs-SCs/CM) were compared by quantifying the expression of markers of pluripotency *OCT4, NANOG*, and *SOX2*, and markers of GCs, *FRAGILIS, STELLA, VASA, DAZL, PIWIL2, SCP3*, and *STRA8* by Q-PCR. The protein expression of *Oct4, Nanog, Dazl*, and *Piwil2* was also analyzed by immunofluorescence. For these experiments, SCs, PB-MSCs, and SSCs cultured with conventional media were used as experimental controls.

### 2.7. Q-PCR Analysis

Quantification of mRNA levels was determined by Q-PCR (Table 1). Total RNA was isolated from the cells using a Quick-RNA MiniPrep (Zymo Research) kit following manufacturer’s instructions. Total RNA was quantified using a Qubit 3.0 (Invitrogen, Fluorometer, CA, USA). DNA genomic digestion was performed using DNAsa I from the Quick-RNA MiniPrep kit (Zymo Research) following the manufacturer’s instructions. The cDNA was synthesized and amplified using a cDNA synthesis kit Q-PCR from Affinity Script (Agilent Technologies, Santa Clara, CA, USA), using a Step One thermal cycler (Applied Biosystems, Foster City, CA, USA). The PCR reaction was performed using a Brilliant SYBR Green QPCR Master Mix kit (Agilent Technologies) and an Eco Real-Time PCR System (Illumina, San Diego, CA, USA). Each reaction tube consists of 5 μL Sybr Green, 1 μL Foward primer, 1 μL Reverse primer, 2 μL nuclease-free H_2_O, and 5 ng cDNA. The amplification of cDNA was extended over 40 cycles, and relative expression analysis was performed using the ΔΔCt (Ct: Threshold Value) method normalized with *GAPDH* and *β-ACTIN* gene expression [32].

### 2.8. Immunofluorescence Analysis

The protein expression of markers for SCs (*Wt1*), SSCs (*Uchl1*), pluripotency (*Oct4* and *Nanog*), MSCs (*Cd73*), and male GCs (*Dazl* and *Piwil2*) was immunodetected in PB-MSCs and SSCs cultured with SCs/CM for 14 days. Culture samples were fixed with 4% PFA at 4 °C for 20 min. Samples were then washed and blocked with 3% bovine serum albumin (BSA) diluted in PBS (pH: 7.4) for 45 min. Proteins were immunodetected using anti-*Wt1* rabbit polyclonal antibody (Cat. # ab89901, Abcam, Boston, MA, USA), anti-*Oct4* mouse polyclonal antibody (Cat. # sc5279, Santa Cruz, CA, USA), anti-*Nanog* mouse polyclonal antibody (Cat. # sc293121, Santa Cruz), anti-*Uchl1* mouse monoclonal antibody (Cat. # 480012, Thermo-Fisher, Waltham, MA, USA), anti-*Cd73* rabbit monoclonal antibody (Cat. # ab137595, Abcam), anti-*Piwil2* rabbit monoclonal antibody (Cat. # ab85084, Abcam), and anti-*Dazl* polyclonal rabbit (Cat. # ab34139, Abcam), diluted in 3% BSA diluted in PBS (pH: 7.4) (*Wt1* 1:200, *Oct4*, *Nanog, Cd73, Piwil2*, and *Dazl* 1:50 and *Uchl1* 1:100). Incubation with primary antibodies was performed overnight at 4 °C. The next day, samples were washed once with PBS and twice with 3% BSA diluted in PBS (pH: 7.4) and incubated in anti-mouse secondary IgG antibody conjugated with Alexa flour 488 (Cat. # A11001, Thermo-Fisher) or FITC-conjugated IgG anti-rabbit antibody (Cat. # ab97050, Abcam) diluted in PBS with BSA 3% (1:500) for 1 h. The samples were then washed again twice at PBS with 3% BSA (pH: 7.4) and once with distilled water, and subsequently mounted on DAPI Fluoroshield medium (Cat. # ab104139, Abcam). Once the protocol was completed, samples were stored at 4 °C protected from light. Samples were imaged using an epifluorescence microscope (Olympus, Shinjuku, Tokyo, Japan) and a spectral confocal microscope (Nikon, Minato-ku, Tokyo, Japan) connected to a digital camera.

### 2.9. Statistical Analysis

The statistical model considered the relative expression of biomarkers as dependent variables and days of culture and type of treatment (PB-MSCs and SSCs cultured in SCs/CM and controls) as independent variables. A value of *p* < 0.05 was used for statistical significance. The significance of the statistical model was analyzed by one-way ANOVA. Differences between the means for relative expression levels between culture days and treatments were determined using Tukey’s post hoc test. The statistical software Infostat 2020 (Cordoba, Argentina) was used in all statistical analyses.

## 3. Results

### 3.1. Morphology of PB-MSCs, SSCs, and SCs Derived from Bull Tissues Isolated by Different Cell Isolation Protocols

**Figure 2:**
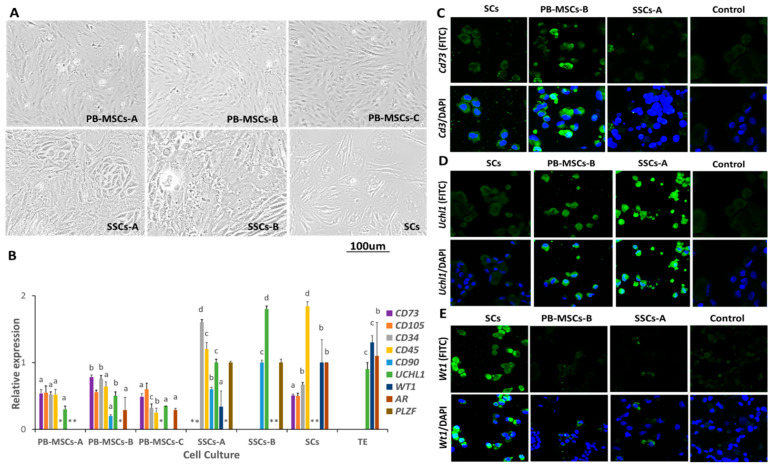
Characterization of SCs, SSCs y PB-MSCs derived from bull tissues. (**A**) PB-MSC-A, PB-MSC-B and PB-MSC-C displayed heterogeneous morphology characterized by circular, fibroblastoid, elongated shapes. SSC-A were adherent, while SSCs-B were both adherent and non-adherent. Morphology of SCs ranged from ovoid to fibroblastoid. (**B**) Relative gene expression of MSCs markers CD73 were higher (*p* < 0.05) in PB-MSCs-B compared to PB-MSCs-A and PB-MSCs-B and was not detected in SSCs-A. CD105 mRNA levels were not different between PB-MSCs-A, PB-MSC-B, PB-MSC-C and SCs, and were not detected in SSCs-A. CD90 gene expression was higher (*p* < 0.05) in SSCs-B and was not detected in PB-MSCs-A and PB-MSCs-C. Gene expression of hematopoietic cell gene marker CD34 was higher (*p* < 0.05) in SSCs-A compared to PB-MSCs-A, PB-MSC-B, PB-MSC-C and SCs. CD45 relative gene expression was higher (*p* < 0.05) in SCs compared to PB-MSCs-A, PB-MSC-B, PB-MSC-C and SSCs-A. UCHL1 gene expression was higher (*p* < 0.05) in SSCs-B and was not detected in SCs, while PLZF was only detect on SSCs and not in PB-MSCs. (**C**) Intense immunofluorescence associated to Cd73 was observed in PB-MSCs-B. (**D**) Immunofluorescence associated to Uchl1 was observed in SSCs-A. (**E**) Intense Wt1 immunofluorescence was observed in SCs. (*) Indicate mRNA levels of specific gene were not detected. Different superscripts (a, b, c, d) indicate differences (*p* < 0.05) for the same marker between cell types.

Morphology of PB-MSCs-A, PB-MSCs-B, and PB-MSCs-C was similar and characterized by circular, fibroblastoid, elongated, and heterogeneous shapes. PB-MSC-B was first to reach confluence (Figure 2A). SSCs-A cultures presented adherent colonies characterized by an irregular and cuboidal morphology, whereas SSCs-B cultures presented both adherent and non-adherent subpopulations. Non-adherent SSCs-B subpopulations presented circular morphology and adherent cells displayed an irregular and cuboidal forms. SC cultures presented diverse morphology that ranged from ovoid, to triangular and fibroblastic with large and well-marked nuclei.

### 3.2. Analysis of Cell-Specific Marker Expression in PB-MSCs, SSCs, and SCs Isolated by Different Protocols

Gene expression of specific markers of MSCs, SSCs, and SCs was determined in all cell types with the aim to determine the purity of primary cell cultures (Figure 2B). *CD73* gene expression was higher (*p* < 0.05) in PB-MSCs-B compared to PB-MSCs-A (0.5 ± 0.06-fold PB-MSCs-B), PB-MSCs-C (0.5 ± 0.05-fold PB-MSCs-B) and SCs (0.5 ± 0.02-fold PB-MSCs-B). *CD73* gene expression was not detected in SSCs-A. Additionally, a cytoplasmic pattern of intense immunofluorescence associated with *Cd73* was observed in PB-MSCs-B (Figure 2C). *CD105* gene expression was not different (*p* > 0.05) between PB-MSCs-A, PB-MSCs-B, and PB-MSCs-C. *CD105* gene expression was not detected in SSCs-A. *CD34* gene expression was higher (*p* < 0.05) in SSCs-A (1.6 ± 0.04-fold PB-MSCs-B) compared to PB-MSCs-A (0.5 ± 0.04-fold PB-MSCs-B), PB-MSCs-B, PB-MSCs-C (0.3 ± 0.06-fold PB-MSCs-B), and SCs (0.7 ± 0.06-fold PB-MSCs). *CD45* gene expression was higher (*p* < 0.05) in SCs (1.8 ± 0.1-fold PB-MSCs-B) compared to SSCs-A (1.2 ± 0.1-fold PB-MSCs-B), PB-MSCs-A (0.55 ± 0.08-fold PB-MSCs-B), PB-MSCs-B, and PB-MSCs-C (0.6 ± 0.07; 0.3 ± 0.02-fold PB-MSCs-B). *CD90* gene expression was higher (*p* < 0.05) in SSCs-B compared to SSCs-A (0.6 ± 0.4-fold SSCs-B) and PB-MSCs-B (0.2 ± 0.02-fold SSCs-B). However, *CD90* gene expression was not detected in PB-MSCs-A, PB-MSCs-C, and SCs. *UCHL1* gene expression was higher (*p* < 0.05) in SSCs-B (1.8 ± 0.4-fold TE) compared to PB-MSCs-A (0.3 ± 0.1-fold TE), PB-MSCs-B (0.5 ± 0.1-fold TE), and PB-MSCs-C (0.4 ± 0.1-fold TE), SSCs-A (1 ± 0.1-fold TE), and TE. *UCHL1* gene expression was not detected in SCs. In addition, intense immunofluorescence associated with *Uchl1* was observed in SSCs-A and PB-MSCs-B (Figure 2D). *WT1* gene expression was higher (*p* < 0.05) in TE (1.3 ± 0.05-fold SCs) compared to SCs and SSCs-A (0.3 ± 0.03-fold SCs) but was not detected in PB-MSCs-A and PB-MSCs-B. In addition, a cytoplasmic pattern of high fluorescence associated with *Wt1* was observed in the SCs (Figure 2E). The gene expression of *AR* was higher (*p* < 0.05) in TE and SCs (1 ± 0.02-fold TE) compared to PB-MSCs-B (0.3 ± 0.2-fold TE) and C (0.35 ± 0.19-fold TE) and was not detected in PB-MSCs-A, SSCs-A, and SSCs-B. *PLZF* gene expression was not different (*p* > 0.05) between SSCs-A and SSCs-B.

### 3.3. Effect of SCs/CM on In Vitro Differentiation of PB-MSCs-B and SSCs-A into GCs

PB-MSCs-B and SSCs-A were selected due to increased expression of specific cell markers and were denominated as PB-MSCs and SSCs for subsequent experiments. To determine the effect of SCs/CM on the in vitro differentiation into GCs of PB-MSCs-B and SSCs-A, both cell types were cultured separately with 50% SCs/CM for 21 days. Next, the expression of pluripotency and GCs genes were evaluated in PB-MSCs and SSCs cultured in SCs/CM during a 21-day culture period.

#### 3.3.1. Morphology of PB-MSCs, SCs, and SSCs Derived from Bull Tissues and Cultured with SCs/CM

**Figure 3:**
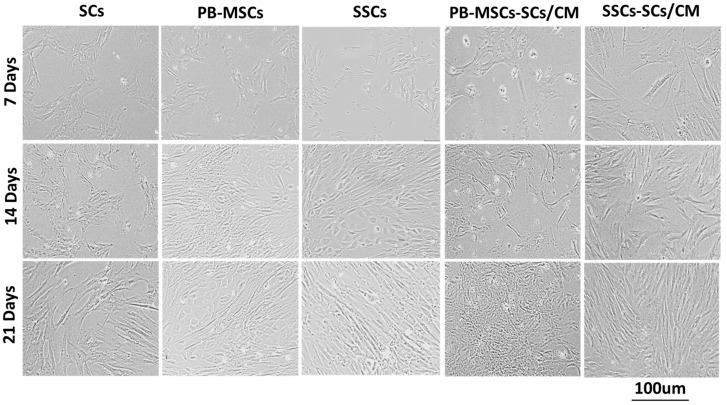
Phase contrast microscopy of single cultures of bull PB-MSCs and SSCs exposed to SCs/CM and controls cultured for 21 days. SCs presented irregular morphology, characterized by fibroblastoid morphology, as became more confluent from days 7 to 21. PB-MSCs presented morphology characterized by circular and fibroblastoid shapes. Adherent SSCs presented circular morphology in suspension and irregular and cuboidal morphology appear in time. Both PB-MSCs or SSCs increased proliferation through the culture period; however, they did not change morphology when cultured with SCs/CM.

SC cultures presented fibroblastoid morphology as cultures became more confluent from days 7 to 21 (Figure 3). PB-MSCs presented morphology characterized by more circular, and elongated shapes. As PB-MSCs advanced in time and confluence, new subpopulations of circular and triangular cells with large nuclei appeared in cultures. SSC cultures presented both adherent and non-adherent subpopulations. Non-adherent subpopulation presented a circular morphology, and adherent cells were characterized by an irregular and cuboidal morphology. Both PB-MSCs and SSCs did not change their morphology when they were cultured with SCs/CM, but increased proliferation, achieving greater confluence on day 14 to 21 of culture in both cell types.

#### 3.3.2. Expression of Pluripotency Markers in PB-MSCs or SSCs Cultured with SCs/CM for 21 Days

**Figure 4:**
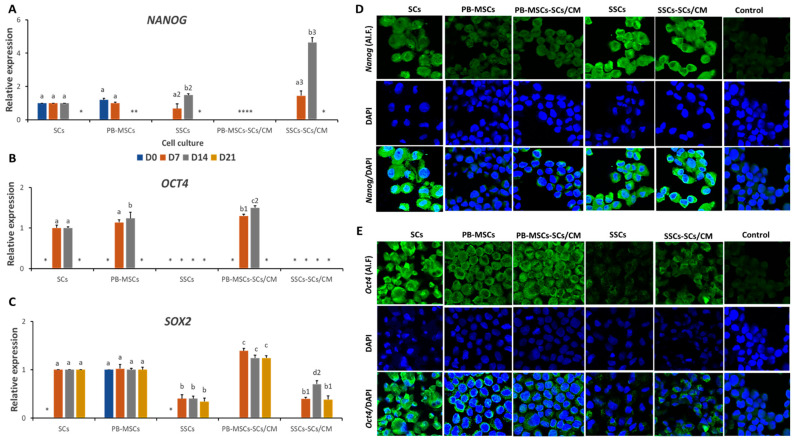
Expression of pluripotency markers NANOG, OCT4 and SOX2 in PB-MSCs and SSCs cultured with SCs/CM for 21 days. (**A**) On days 7 and 14, NANOG gene expression was higher (*p* < 0.05) in SSCs-SCs/CM, whereas no NANOG gene expression was detected in PB-MSCs-SCs/CM. (**B**) OCT4 gene expression was higher (*p* < 0.05) in PB-MSCs-SCs/CM on day 7 and 14 compared to SCs and PB-MSCs control. Gene expression of OCT4 was not detected in SSCs and SSCs-SCs/CM. (**C**) *SOX2* gene expression of was higher (*p* < 0.05) on days 7, 14, and 21 in PB-MSCs-SCs/CM compared to PB-MSCs, SSCs-SCs/CM and SSCs. (**D**) Immunofluorescence associated with *Nanog* was intense in SCs, SSCs, and SSCs-SCs/CM on day 14. (**E**) intense immunofluorescence associated with *Oct4* was observed in SCs, PB-MSCs, and PB-MSCs-SCs/CM and on day 14. (*) Indicate mRNA levels of specific gene were not detected. Different superscripts (a, b, c, d) indicate differences (*p* < 0.05) between cell types for specific day of culture and (1,2,3) for the same marker between cell types.

Gene expression of *NANOG* was not different (*p* > 0.05) between SCs and PB-MSCs (1.2 ± 0.09-fold SCs) on day 0 of culture (Figure 4A, Table 2). Gene expression of *NANOG* increased (*p* < 0.05) on days 7 and 14 of culture in the SSCs-SCs/CM (1.4 ± 0.3 and 4.6 ± 0.2-fold SCs, respectively) compared to SSCs (0.7 ± 0.3- and 1.5 ± 0.1-fold SCs, respectively). Gene expression of *NANOG* was not detected in PB-MSCs-SCs/CM in any day of the culture. Immunofluorescence associated with *Nanog* was intense in SCs, SSCs, and SSCs-SCs/CM on day 14 (Figure 4D, Table 2). Gene expression of *OCT4* was higher (*p* < 0.05) in the PB-MSCs-SCs/CM on days 7 and 14 (1.3 ± 0.04- and 1.5 ± 0.05-fold SCs) compared to PB-MSCs (1 ± 0.04- and 1.2 ± 0.06-fold SCs) and SCs. Gene expression of *OCT4* was not detected in SSCs or SSCs-SCs/CM in any day of culture (Figure 4B, Table 2). Additionally, intense immunofluorescence associated with *Oct4* was observed in SCs, PB-MSCs, and PB-MSCs-SCs/CM and on day 14 of culture (Figure 4E, Table 2). Gene expression of *SOX2* was higher (*p* < 0.05) on days 7, 14, and 21 in PB-MSCs-SCs/CM (1.4 ± 0.08-, 1.2 ± 0.05-, and 1.2 ± 0.07-fold SCs, respectively) compared to PB-MSCs (1 ± 0.09-, 1 ± 0.03-, and 1 ± 0.05-fold SCs, respectively), SSCs-SCs/CM (0.4 ± 0.03-, 0.7 ± 0.07-, and 0.4 ± 0.08-fold SCs) and SSCs (0.4 ± 0.08-, 0.4 ± 0.05-, and 0.3 ± 0.07-fold SCs) (Figure 4C, Table 2).

#### 3.3.3. Expression of GCs Markers in PB-MSCs or SSCs Cultured with SCs/CM for 21 Days

**Figure 5:**
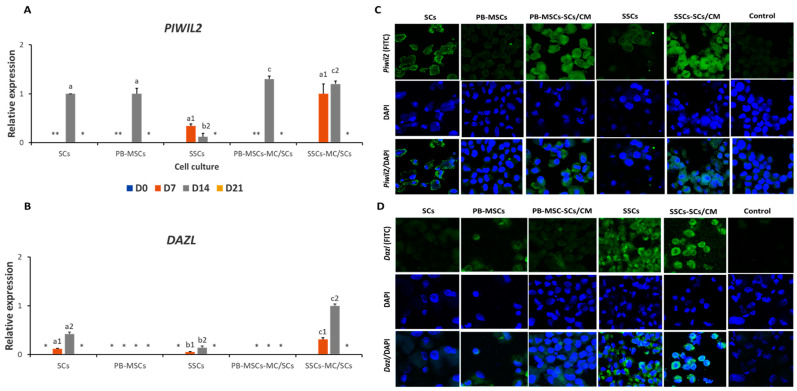
Expression of GCs markers PIWIL2 and DAZL in PB-MSCs and SSCs treated with SCs/CM for 21 days. (**A**) Higher (*p* < 0.05) gene expression of PIWIL2 was detected in the SSCs-SCs/CM on day 7. On day 14 of culture, gene expression of PIWIL2 was higher (*p* < 0.05) in PB-MSCs-SCs/CM and SSCs-SCs/CM compared to SCs, PB-MSCs and SSCs controls. Over time the relative expression of PIWIL2 increased (*p* < 0.05) in SSCs-SCs/CM from day 7 to day 14. (**B**) The relative expression of DAZL was higher (*p* < 0.05) in SSCs-SCs/CM on days 7 and 14 compared with SCs and SSCs controls. In PB-MSCs or PB-MSCs-SCs/CM no gene expression of DAZL was detected. (**C**) *Piwil2* protein expression was immunodetected in PB-MSCs-SCs/CM and SSCs-SCs/CM on day 14. (**D**) *Dazl* was immunodetected in SSCs and SSCs-SCs/CM on day 14. (*) Indicate mRNA levels of specific gene were not detected. Different superscripts (a, b, c) indicate differences (*p* < 0.05) for the same marker between cell types and (1,2) for the same marker between days of culture.

*PIWIL2* gene expression was higher (*p* < 0.05) in SSCs-SCs/CM (1.00 ± 0.2-fold) compared to SSCs (0.34 ± 0.04-fold SSCs-SCs/CM) on day 7 (Figure 5A). On day 14, *PIWIL2* gene expression increased (*p* < 0.05) in PB-MSCs-SCs/CM (1.3 ± 0.05-fold SCs) and SSCs-SCs/CM (1.2 ± 0.04-fold SCs) cultures compared to PB-MSCs (1.00 ± 0.043-fold SCs) and SSCs (0.1 ± 0.07-fold SCs). Relative gene expression of *PIWIL2* in SSCs-SCs/CM increased (*p* < 0.05) from day 7 (1.0 ± 0.2-fold SSCs) to day 14 (1.2 ± 0.04-fold SCs). Additionally, *Piwil2* protein expression was immunodetected in PB-MSCs-SCs/CM and SSCs-SCs/CM on day 14 (Figure 5C, Table 2). Relative gene expression of *DAZL* was higher (*p* < 0.05) in SSCs-SCs/CM on days 7 (0.31 ± 0.03-fold SCs) and 14 (1.00 ± 0.04-fold SCs) compared with SCs and SSCs (0.05 ± 0.01- and 0.14 ± 0.03-fold SCs, respectively) (Figure 5B, Table 2). *DAZL* gene expression increased (*p* < 0.05) from day 7 to 14 in SCs (0.12 ± 0.03- and 0.42 ± 0.04-fold SCs), SSCs (0.05 ± 0.01- and 0.14 ± 0.03-fold SCs) and SSCs (0.31 ± 0.03 and 1-fold SCs). However, mRNA levels of DAZL were not detected in PB-MSCs or in PB-MSCs-SCs/CM. Intense labelling associated with *Dazl* was immunodetected in SSCs and SSCs-SCs/CM on day 14 (Figure 5D, Table 2). Gene expression of CG markers *FRAGILIS*, *STELLA*, *VASA, SCP3*, and *STRA8* was also analyzed by *Q-PCR*; however, mRNA levels of these genes were not detected in any cell type.

#### 3.3.4. Expression of MSCs Markers in PB-MSCs or SSCs Cultured with SCs/CM for 21 Days

**Figure 6:**
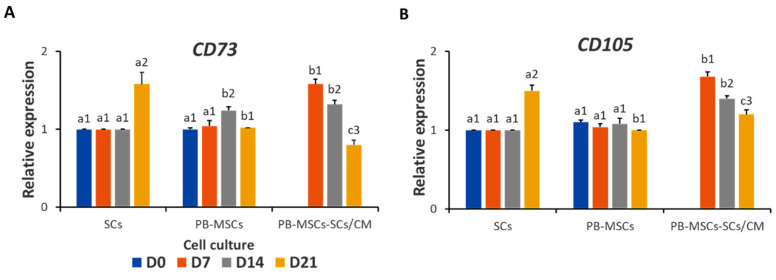
Relative gene expression of MSCs markers CD73 and CD105 in PB-MSCs cultured with SCs/CM for 21days. (**A**) CD73 gene expression was higher (*p* < 0.05) on days 7 and 14 in PB-MSCs-SCs/CM and SCs on day 21. Over time, CD73 gene expression in PB-MSCs-SCs/CM decreased (*p* < 0.05). (**B**) The relative expression of CD105 was higher (*p* < 0.05) on days 7 and 14 in the culture of PB-MSCs- SCs/CM and in SCs on day 21. Over time, the relative expression of CD105 in PB-MSCs-SCs/CM decreased (*p* < 0.05). Different superscripts (a, b, c) indicate differences (*p* < 0.05) for the same marker between cell types and (1,2,3) for the same marker between days of culture.

*CD73* and *CD105* gene expression increased (*p* < 0.05) in PB-MSCs-SCs/CM (1.6 ± 0.04- and 1.3 ± 0.05-fold SCs) compared with controls PB-MSCs (1.0 ± 0.04- and 1.1 ± 0.20-fold SCs) and SCs on day 7 (Figure 6A, B; Table 2). *CD73* and *CD105* gene expression also increased (*p* < 0.05) in PB-MSCs-SCs/CM (1.7 ± 0.05- and 1.4 ± 0.04-fold SCs) compared with controls PB-MSCs (1.0 ± 0.04- and 1.1 ± 0.20-fold SCs) and SCs on day 14. In comparison, the expression of *CD73* and *CD105* decreased (*p* < 0.05) in PB-MSCs-SCs/CM (0.8 ± 0.04-fold PB-MSCs and 1.2 ± 0.02-fold PB-MSCs) compared with SCs (1.6 ± 0.4-fold PB-MSCs and 1.5 ± 0.07-fold PB-MSCs) and PB-MSCs on day 21.

## 4. Discussion

Given the potential of MSC for transdifferentiation, previous studies have focused on the generation of GCs from MSCs using different in vitro differentiation approaches including co-culture systems with SCs [10,12,13,14,16]. Cell co-culture systems can largely simulate the in vivo environment, and they facilitate an assessment of the interaction between different types of cells and their environment. These cultures systems also permit the exploration of the mechanism of action of effector cells over target cells and their potential targets. However, cell co-culture systems have the disadvantage that cells are mixed in culture, which makes it difficult to study specific cell populations. This drawback may be improved by using CM of effector cells, which contain the specific molecules that mediate their effect, and allow the homogeneity of target cells to be maintained [14,33]. In the present study, we compared the potential of bull PB-MSCs and SSCs for GC differentiation using 2D culture systems that included exposure to 50% SCs/CM during a 21-day culture period.

MSCs were isolated from bovine PB by three different protocols that included conventional separation in Percoll gradient (PB-MSCs-A), separation in Histopaque gradient (PB-MSCs-B), and sequential centrifugation in two concentrations of Percoll (1.053 and 1.064 g/mL) (PB-MSCs-C). Among these protocols, Histopaque separation (PB-MSCs-B) yielded cultures with higher levels of expression of mesenchymal marker *CD73*, which has been reported as a specific marker for isolation of MSCs [1,4,10,12,13]. Nevertheless, cells isolated using this protocol also expressed high levels of hematopoietic markers CD34 and CD45, which indicate that a high proportion of blood cells were included in the cell pool. Subsequent plastic-adherence separation and morphological evaluation allowed the removal of hematopoietic cells from PB-MSC cultures. Thus, according to these analyses, Histopaque-based separation was the most efficient protocol for the isolation of bull PB-MSCs.

PB is an interesting source for obtaining MSCs due to numerous comparative advantages over other sources, including the availability of donors of different ages and sexes, the absence of ethical concerns and the minimally invasive collection procedure [6,8,13]. Despite the trilineage mesodermal differentiation capacity (osteogenic, adipogenic and chondrogenic) of bovine PB-MSCs not having been previously described, previous studies have indicated that PB-MSCs from different species including human, feline and ovine models have mesodermal and non-mesodermal differentiation potential [34,35,36].

Cultures of SCs used in the present study were isolated from abattoir-derived testis and were selected and expanded as previously reported [12,13]. Isolated bull SCs expressed high mRNA levels of *WT1* and *AR*, and displayed intense immunofluorescence associated with *Wt1*. Our previous studies have reported a positivity of around 90% for *Wt1* in SCs isolated by this protocol and analyzed by flow cytometry [12]. Both *WT1* and *AR* markers have previously been identified as specific for SCs in murine, human, and bovine [12,13,37,38]. *Wt1* has been associated with gonad development and acts as a transcription factor that regulates the inductive signal from the mesenchyme to the coelomic epithelium of the mesonephro by controlling the growth of gonadal ridges that give rise to SCs [39]. *AR* functions as a nuclear receptor and as a ligand-dependent transcription factor, which regulates the expression of a wide variety of genes involved in the development of puberty and male fertility [40]. Our results indicate that SC cultures isolated by this protocol were highly homogenous and suitable for CM production and for subsequent GC differentiation experiments.

Bull SSCs used in this study (SSCs-A) were isolated by two-step enzymatic digestion, sequential filtration and differential adhesion following a previously reported protocol [30]. Although no consistent bull SSCs markers have been determined, *UCHL1* and *PLZF* have been specifically detected in bull SSCs, as reported in several studies [13,40,41,42]. *UCHL1* is related to the events of self-renewal and colocalization of SSCs and is specifically expressed in vivo in SSCs located in the basement membrane of seminiferous tubules, but not in differentiated GCs [43,44]. *PLZF* is a conserved marker of undifferentiated GCs in animal model including cattle, sheep, and goats [45,46]. *PLZF* is a transcriptional factor that represses the transcription of c-kit, which is a hallmark of SSC differentiation and thereby maintains the source of SSCs [47]. In addition, CD90-positive cells have been shown to exhibit cardinal properties of SSCs, including proliferation, differentiation, and colony formation in human and mouse models [48,49]. Isolated SSCs populations used in this study expressed high levels of *UCHL, PLZF*, and *CD90* mRNA and were immunopositive for *Uchl1*, which indicates that our protocol yielded consistent and homogeneous SSC cultures. Previous studies have indicated that SSCs derived from domestic animals can be easily isolated and cultivated; however, they undergo a gradual decrease in proliferation during subsequent subcultures, and over time, spontaneous differentiation and apoptosis dominate cellular events, with the eventual arrest of proliferation [13,50]. In this context, the expansion of SSCs under in vitro conditions can only be maintained for less than 60 days, due to spontaneous differentiation towards nonspecific cell lineage [42,51,52,53]. However, primary cultures of SSCs used in our study were kept in culture for intervals not exceeding 30 days, and during this time, SSCs maintained continuous proliferation, and were adherent to plastic with irregular and cuboidal morphology. In addition, these bull SSCs were able to respond to culture with SCs/CM suggesting they did not undergo spontaneous differentiation.

PB-MSCs-SCs/CM and SSCs-SCs/CM displayed greater confluence on day 21 of culture in both cell types with little changes in morphology. SSCs-SCs/CM increased *NANOG* expression on day 14 and decreased *SOX2* expression, while PB-MSCs-SCs/CM increased *OCT4* and *SOX2* expression on days 7 and 14. Moreover, SSCs-SCs/CM did not express *OCT4* and PB-MSCs-SCs/CM lost *NANOG* expression during differentiation. *NANOG*, *OCT4*, and *SOX2* are transcription factors that regulate common target genes that promote pluripotency and self-renewal, while inhibiting the differentiation processes [54]. *NANOG* expression is promoted by *OCT4* and *SOX2* expression [54,55], and together, these three factors regulate a network of genes involved in control of factors associated with chromatin remodeling, cell cycle, and signal suppression [56]. These genes are expressed in both pluripotent GCs and early GCs [56,57]. Consequently, its high expression indicates a state of undifferentiation in pluripotent cells, and its low expression would occur during cell differentiation [58,59]. In the present study, the absence of *OCT4* and the increased expression of *NANOG* seems to play a role in SSC differentiation, whereas the absence of *NANOG* and the increased role of *OCT4* may be required for PB-MSC differentiation into GCs. Despite OCT4, NANOG, and SOX2 regulating the maintenance of pluripotent state in embryos and derived cells in most mammalian species, their roles in bovine MSC has not been completely determined. However, recent reports indicate that concomitant ectopic expression of OCT4, SOX2, and KLF4 and c-MYC is able to induce formation of bovine induced pluripotent stem cells (iPSCs) suggesting an important role in the control of pluripotency [60]. Considering this potential role, we may speculate that KLF4 and c-MYC may also participate in MSC differentiation and be regulated under the effect of SCs/CM. Thus, our results indicate that exposure to SCs/CM induced distinctive and contrasting expression patterns of pluripotency markers in bull PB-MSCs or SSCs during differentiation into GCs.

Exposure to SCs/CM increased the expression of *PIWIL2* and *DAZL* in SSCs and only increased the expression of *PIWIL2* in PB-MSCs. *DAZL* is essential for mammalian spermatogenesis and is involved in proliferation, development, maturation, and functional maintenance of male GCs [61]. *PIWIL2* is exclusively expressed in GCs, where it has a role in self-renewal of SSCs [62,63]. In the mouse, *PIWIL2* null mutants have been shown to have incomplete spermatogenesis and cannot produce sperm [62]. The results obtained in this study indicate that SSCs cultured with SCs/CM increased the expression of a greater number of GC genes compared to PB-MSCs cultured under the same condition. The more restricted GCs profile of PB-MSCs may be associated with its multipotency capacity associated with less specific mesodermal lineages including osteogenic, adipogenic and chondrogenic. In comparison, the unipotent differentiation ability of SSCs towards GCs and sperm allows them to display a more robust GC expression pattern during the differentiation process. Previous studies from our group have reported that AT-MSCs and BM-MSCs, co-cultured with SCs in 2D systems, increased the expression of *DAZL* and *PIWIL2* [12]. Moreover, we found that SSCs and PB-MSCs acquired a GC profile after 14 days in 3D co-culture system with SCs [13]. In addition, PB-MSCs-SCs/CM lose mesenchymal profile on day 21 by lowering the expression of *CD73* and *CD105*. This loss of mesenchymal profile has been previously reported in BM-MSCs and AT-MSCs after 14 days of culture with SCs in adherent monolayer [12].

In the present study, gene expression of GC markers *VASA, FRAGILIS, STELLA, SCP3*, and *STRA8* were not detected in any cell type or treatment. The expression of *VASA, STELLA, SCP3*, and *STRA8* has been related to later stages of GC maturation including post-migratory phase [61,63]. *VASA* gene encodes an RNA-binding protein and an ATP-dependent RNA helicase present in meiotic GCs and associated with the appearance of chromatoid bodies in spermatocytes and spermatids [46,63]. *STELLA* is believed to be involved in chromosomal organization and RNA processing and its expression has been detected during blastocyst stage after which it disappears, suggesting that *STELLA* is apparently not necessary for germline development [63]. *SCP3* is a meiosis-specific supra molecular proteinaceous structure which functions as a molecular framework that regulates chromosome synapsis [64]. Consequently, the lack of *SCP3* expression suggest that PB-MSCs and SSCs did not undergo progression into meiosis under the current conditions. *STRA8* is a key factor involved in meiotic entry of postmigratory PGCs, in a process regulated by RA [65]. *FRAGILIS* is an early primordial marker of GCs, expressed in response to BMP4 signaling from the extra-embryonic ectoderm [63]. Nevertheless, the requirement of RA and BMP4 activation and the subsequent lack of STRA8 and FRAGILIS gene expression suggest that SCs/CM may have not achieved sufficient levels of these factor or its dilution with 50% standard media reduced its effective concentrations.

## 5. Conclusions

Considering the increased levels of *CD73* gene expression and the subsequent separation of hematopoietic cells, a histopaque-based protocol was shown to be the most efficient for the isolation of bull PB-MSCs. Exposure to SCs/CM induced distinctive and contrasting expression patterns of pluripotency markers in bull PB-MSCs or SSCs. The absence of *OCT4* and the increased expression of *NANOG* seems to play a role in SSC differentiation, whereas the absence of *NANOG* and the increased role of *OCT4* may be required for PB-MSC differentiation into GCs. SSCs cultured with SCs/CM increased the expression of *PIWIL2* and *DAZL*, while PB-MSCs cultured under the same condition only increased the expression of *DAZL*. Overall, the pattern of gene expression reported in these results associated with pluripotent and GCs markers suggests that PB-MSCs and SSCs activate different signaling pathways after exposure to SCs/CM and during differentiation into GCs.

## Figures and Tables

**Table 1:** Sequence of primers used for Q-PCR analysis.

Genes	Nucleotide sequence (5′-3′)		*n.*Access
	Forward	Reverse	
Endogenous genes			
*β- ACTINA*	CGCACCACTGGCATTGTCAT	TCCAAGGCGACGTAGCAGAG	NM_173979.3
*GAPDH*	CCTTCATTGACCTTCACTACATGG TCTA	TGGAAGATGGTGATGGCCTTTCCATTG	NM_001034034.2
Mesenchymal stem cell genes			
*CD73*	TGGTCCAGGCCTATGCTTTTG	GGGATGCTGCTGTTGAGAAGAA	NM_174129.3
*CD105*	CGGACAGTGACCGTGAAGTTG	TGTTGTGGTTGGCCTCGATTA	NM_00107639.1
Hematopoietic cell genes			
*CD34*	CATGCCGTCTTAACCCATCT	CGGTCTACAGAGGTGGTGGT	NM_174009.1
*CD45*	CCACGGGTATTCAGCAAGTT	CCCAGATCATCCTCCAGAAA	NM_001206523
Sertoli cell genes			
*WT1*	CGTGCGTACCATGTAGGGAA	CTCGTGCTTGAAGGAGTGGT	XM_015474834.2
*AR*	CAGATGGCAGTCATTCAG	CTTGGTGAGCTGGTAGAAG	XM_001244127
Spermatogonial stem cell genes			
*UCHL1*	AGAAGCAGCATCTCGGTTCC	CGTGGTTGAGGGTAAGTGCT	NM_001046172.2
*PLZF*	GCCGTGATACCGAGAGCAAC	CTCTCCTCGCTGGAATGCTT	NM_001037476.2
*CD90*	ACTCATACCGCTCCCGAACCA	CATGTGTATGTCCCCTCGTCCTT	NM_001034765
Germ cell genes			
*DAZL*	TCCAAGTTCACCAGTTCAGG	CGT CTG TAT GCT TCT GTC CAC	NM_001081725.1
*STRA8*	TGTGCCCAGGTGTTCATCTC	GGGGACTGTCACCTCATTGG	XM_015463130
*PIWIL2*	TCGTATTGATGATGTGGATTGG	GGGAGCAGCAGGATTTCAC	XM_617223.3
*FRAGILIS*	ATCTGCAGCGAGACCTCTGT	CCGATGGACATGATGATGAG	XM_002697323
*STELLA*	TGCAAGTTGCCACTCAACTC	TCTTACCCCTCTCCGCCTAT	NM_00111110
*VASA*	TGCTACTCCTGGAAGACTGA	CGGTCTGCTGAACATCTCTA	NM_001007819.1
*SCP3*	CTAGAATTGTTCAGAGCCAGAG	GTTCAAGTTCTTTCTTCAAAG	NM_001040588.2
Pluripotency genes			
*OCT4*	GAAAGAGAAAGCGGACGAG	GTGAAAGGAGACCCAGCAG	NM_174580.2
*NANOG*	TAAGCACAGGGGGCAAAAGT	ATGGCTAAAAGGGGTGGAGG	NM_001025344.1
*SOX2*	CCCGTGGTTACCTCTTCTTCC	CGCTCTGCTAGTGCTGGGAC	NM_001105463.2

**Table 2:** Expression pattern of mesenchymal, pluripotency and germline markers in cultured cattle PB-MSCs/CM and SSCs/SCs compared to their controls for PB-MSCs and SSCs.

Marker	PB-MSCs-SCs/CM	SSCs- SCs/CM
*CD73*	↑ (Q-PCR)	-
*CD105*	↑ (Q-PCR)	-
*DAZL*	x (Q-PCR)	↑ (Q-PCR) *(IF)
*STRA8*	x (Q-PCR)	x (Q-PCR)
*PIWIL2*	↑ (Q-PCR) *(IF)	↑ (Q-PCR) *(IF)
*FRAGILIS*	x (Q-PCR)	x (Q-PCR)
*STELLA*	x (Q-PCR)	x (Q-PCR)
*OCT4*	↑ (Q-PCR) *(IF)	x (Q-PCR) *(IF)
*NANOG*	x (Q-PCR)	↑ (Q-PCR) *(IF)
*SOX2*	↑ (Q-PCR)	↑ (Q-PCR)
*SCP3*	x (Q-PCR)	x (Q-PCR)
*VASA*	x (Q-PCR)	x (Q-PCR)

Abbreviations: ↑, increase expression; x, not detected; - not evaluated; *, immunodetected; Q-PCR, quantitative PCR; IF, Immunofluorescence.

## Data Availability

The data presented in this study are available on request from the corresponding author.

## References

[1] Dominici M.L.B.K., Le Blanc K., Mueller I., Slaper-Cortenbach I., Marini F.C., Krause D.S., Horwitz E.M. (2006). Minimal criteria for defining multipotent mesenchymal stromal cells. The International Society for Cellular Therapy position statement. Cytotherapy.

[2] Oliveira É.M.D. (2013). Diferenciação de Células-Tronco em Hepatócitos e Desenvolvimento de Modelo Pré-Clínico de Fibrose Hepática para Ensaios de Terapia Celular. Ph.D. Thesis.

[3] Kohyama J., Abe H., Shimazaki T., Koizumi A., Okano H., Hata J., Gojo S. (2001). Brain from bone: Efficient “meta-differentiation” of marrow stroma-derived mature osteoblasts to neurons with Noggin or a demethylating agent. Differentiation.

[4] Dueñas F., Becerra V., Cortes Y., Vidal S., Sáenz L., Palomino J., Peralta O.A. (2014). Hepatogenic and neurogenic differentiation of bone marrow mesenchymal stem cells from abattoir-derived bovine fetuses. BMC Vet. Res..

[5] Vater C., Kasten P., Stiehler M. (2011). Culture media for the differentiation of mesenchymal stromal cells. Acta Biometer..

[6] Li S., Huang K.J., Wu J.C., Hu M.S., Sanyal M., Hu M., Logaker H.Y., Lorenz H.P. (2015). Peripheral blood-derived mesenchymal stem cells: Candidate cells responsible for healing critical-sized calvarial bone defects. Stem Cells Transl. Med..

[7] Calle A., Gutiérrez-Reinoso M.Á., Re M., Blanco J., De La Fuente J., Monguió-Tortajada M., Ramírez M.Á. (2021). Bovine peripheral blood MSC chemotax towards inflammation and embryo implantation stimuli. J. Cell. Physiol..

[8] Lin W., Xu L., Lin S., Shi L., Wang B., Pan Q., Li G. (2019). Characterisation of multipotent stem cells from human peripheral blood using an improved protocol. J. Orthop. Translat..

[9] He Q., Wan C., Li G. (2007). Concise review: Multipotent mesenchymal stromal cells in blood. Stem Cells.

[10] Cortez J., Bahamonde J., De Los Reyes M., Palomino J., Torres C.G., Peralta O.A. (2018). In vitro differentiation of bovine bone marrow-derived mesenchymal stem cells into male germ cells by exposure to exogenous bioactive factors. Reprod. Domest. Anim..

[11] Cordero P., Guerrero-Moncayo A., De Los Reyes M., Varas-Godoy M., Cortez J., Torres C.G., Peralta O.A. (2021). Overexpression of DAZL, STRA8, and BOULE genes and treatment with BMP4 or retinoic acid modulate the expression of MSC overexpressing germ cell genes. Front. Vet. Sci..

[12] Segunda M.N., Bahamonde J., Muñoz I., Sepulveda S., Cortez J., De Los Reyes M., Peralta O.A. (2019). Sertoli cell-mediated differentiation of bovine fetal mesenchymal stem cells into germ cell lineage using an in vitro coculture system. Theriogenology.

[13] Segunda M.N., Díaz C., Torres C.G., Parraguez V.H., De los Reyes M., Peralta O.A. (2023). Comparative Analysis of the Potential for Germ Cell (GC) Differentiation of Bovine Peripheral Blood Derived-Mesenchymal Stem Cells (PB-MSC) and Spermatogonial Stem Cells (SSC) in Co-Culture System with Sertoli Cells (SC). Animals.

[14] Wei X., Peng G., Zheng S., Wu X. (2012). Differentiation of umbilical cord mesenchymal stem cells into steroidogenic cells in comparison to bone marrow mesenchymal stem cells. Cell Prolif..

[15] Ghaem Maghami R., Mirzapour T., Bayrami A. (2018). Differentiation of mesenchymal stem cells to germ-like cells under induction of Sertoli cell-conditioned medium and retinoic acid. Andrologia.

[16] Xie L., Lin L., Tang Q., Li W., Huang T., Huo X., Ma L. (2015). Sertoli cell-mediated differentiation of male germ cell-like cells from human umbilical cord Wharton’s jelly-derived mesenchymal stem cells in an in vitro coculture system. Eur. J. Med. Res..

[17] Zhang D., Liu X., Peng J., He D., Lin T., Zhu J., Li X., Zhang Y., Wei G. (2014). Potential spermatogenesis recovery with bone marrow mesenchymal stem cells in an azoospermic rat model. Int. J. Mol. Sci..

[18] Jabari A., Gilani M.A.S., Koruji M., Gholami K., Mohsenzadeh M., Khadivi F., Movassagh S.A. (2020). Three-dimensional coculture of human spermatogonial stem cells with Sertoli cells in soft agar culture system supplemented by growth factors and Laminin. Acta Histochem..

[19] Monteiro C.D., Bicudo S.D., Toma H.S. (2010). O papel das células de Sertoli na espermatogênese. Pubvet.

[20] Lie P.P., Cheng C.Y., Mruk D.D. (2013). Signaling pathways regulating the blood—Testis barrier. Int. J. Biochem. Cell Biol..

[21] Huleihel M., Nourashrafeddin S., Plant T.M. (2015). Application of three-dimensional culture systems to study mammalian spermatogenesis, with an emphasis on the rhesus monkey (*Macaca mulatta*). Asian J. Androl..

[22] Diaz T., Marta A. (2016). Impacto de la Nutrición Diferencial Durante la Preñez y Lactancia Sobre el Factor Neurotrófico Derivado de células Gliales (GDNF) en Testículos de Ratas Adultas. PhD Thesis.

[23] Monfared M.H., Minaee B., Rastegar T., Khrazineiad E., Barbarestani M. (2016). Sertoli cell condition medium can induce germ like cells from bone marrow derived mesenchymal stem cells. Iran. J. Basic Med. Sci..

[24] Mohammadzadeh E., Mirzapour T., Nowroozi M.R., Nazarian H., Piryaei A., Alipour F., Ghaffari Novin M. (2019). Differentiation of spermatogonial stem cells by soft agar three-dimensional culture system. Artif. Cells Nanomed. Biotechnol..

[25] Luo Y., Xie L., Mohsin A., Ahmed W., Xu C., Peng Y., Guo M. (2019). Efficient generation of male germ-like cells derived during co-culturing of adipose-derived mesenchymal stem cells with Sertoli cells under retinoic acid and testosterone induction. Stem Cell Res. Ther..

[26] Nicholls P.K., Schorle H., Naqvi S., Hu Y.C., Fan Y., Carmell M.A., Page D.C. (2019). Mammalian germ cells are determined after PGC colonization of the nascent gonad. Proc. Natl. Acad. Sci. USA.

[27] Pawitan J.A. (2014). Prospect of stem cell conditioned medium in regenerative medicine. BioMed Res. Int..

[28] Nguyen L.T., Tran N.T., Than U.T.T., Nguyen M.Q., Tran A.M., Do P.T.X., Hoang N.T.M. (2022). Optimization of human umbilical cord blood-derived mesenchymal stem cell isolation and culture methods in serum-and xeno-free conditions. Stem Cell Res. Ther..

[29] Harms C.A., Keller J., Kennedy-Stoskopf S. (2000). Use of a two-step Percoll^®^ gradient for separation of loggerhead sea turtle peripheral blood mononuclear cells. J. Wildl Dis..

[30] Ganan-Gomez I., Clise-Dwyer K., Colla S. (2022). Isolation, culture, and immunophenotypic analysis of bone marrow HSPCs from patients with myelodysplastic syndromes. STAR Protoc..

[31] Hamidabadi H.G., Bojnordi M.N. (2018). Coculture of mouse spermatogonial stem cells with sertoli cell as a feeder layer, stimulates the proliferation and spermatogonial stemness profile. Middle East Fertil. Soc. J..

[32] Schefe J.H., Lehmann K.E., Buschmann I.R., Unger T., Funke-Kaiser H. (2006). Quantitative real-time RT-PCR data analysis: Current concepts and the novel “gene expression’s C T difference” formula. J. Mol. Med..

[33] Liu R., Meng X., Yu X., Wang G., Dong Z., Zhou Z., Wang F. (2022). From 2D to 3D co-culture systems: A review of co-culture models to study the neural cells interaction. Int. J. Mol. Sci..

[34] Trivanović D., Kocić J., Mojsilović S., Krstić A., Ilić V., Djordjević I.O., Santibanez J.F., Jovcić G., Terzić M., Bugarski D. (2013). Mesenchymal stem cells isolated from peripheral blood and umbilical cord Wharton’s jelly. Srp. Arh. Celok. Lek..

[35] Sato K., Yamawaki-Ogata A., Kanemoto I., Usui A., Narita Y. (2016). Isolation and characterisation of peripheral blood-derived feline mesenchymal stem cells. Vet J..

[36] Lyahyai J., Mediano D.R., Ranera B., Sanz A., Remacha A.R., Bolea R., Martín-Burriel I. (2012). Isolation and characterization of ovine mesenchymal stem cells derived from peripheral blood. BMC Vet. Res..

[37] Chen M., Zhang L., Cui X., Lin X., Li Y., Wang Y., Gao F. (2017). Wt1 directs the lineage specification of sertoli and granulosa cells by repressing Sf1 expression. Development.

[38] Wang W.J., Wu S.P., Liu J.B., Shi Y.S., Huang X., Zhang Q.B., Yao K.T. (2013). MYC regulation of CHK1 and CHK2 promotes radio resistance in a stem cell-like population of nasopharyngeal carcinoma cells. Cancer Res..

[39] Piprek R.P., Kloc M., Kubiak J.Z. (2016). Early development of the gonads: Origin and differentiation of the somatic cells of the genital ridges. Molecular Mechanisms of Cell Differentiation in Gonad Development.

[40] Zheng Y., Zhang Y., Qu R., He Y., Tian X., Zeng W. (2014). Spermatogonial stem cells from domestic animals: Progress and prospects. Reproduction.

[41] Fujihara M., Kim S.M., Minami N., Yamada M., Imai H. (2011). Characterization and in vitro culture of male germ cells from developing bovine testis. J. Reprod. Dev..

[42] Herrid M., Davey R.J., Hill J.R. (2007). Characterization of germ cells from pre-pubertal bull calves in preparation for germ cell transplantation. Cell Tissue Res..

[43] Luo J., Megee S., Dobrinski I. (2009). Asymmetric distribution of UCH-L1 in spermatogonia is associated with maintenance and differentiation of spermatogonial stem cells. J. Cell. Physiol..

[44] Ram K., Kannan T., Basha S., Geetha Ramesh G.R., William B. (2017). In-vitro culture morphology of Spermatogonial stem cells (SSCs) in mice. Int. J. Livest. Res..

[45] Bahadorani M., Hosseini S.M., Abedi P., Hajian M., Afrough M., Azhdari T.Z., Nasr-Esfahani M.H. (2011). Comparative immunohistochemical analysis of VASA, PLZF and THY1 in goats and sheep suggests that these markers are also conserved in these species. J. Cytol. Histol..

[46] Nabulindo N.W., Nguhiu-Mwangi J., Kipyegon A.N.E., Ogugo M., Muteti C., Christian T., Kemp S. (2022). Culture of Kenyan goat (Capra hircus) undifferentiated spermatogonia in feeder-free conditions. Front Vet Sci..

[47] Costoya J.A., Hobbs R.M., Barna M., Cattoretti G., Manova K., Sukhwani M., Pandolfi P.P. (2004). Essential role of Plzf in maintenance of spermatogonial stem cells. Nat. Genet..

[48] Zhao Y., Ye S., Liang D., Wang P., Fu J., Ma Q., Wang Y. (2018). In vitro modeling of human germ cell development using pluripotent stem cells. Stem Cell Rep..

[49] Li N., Ma W., Shen Q., Zhang M., Du Z., Wu C., Hua J. (2019). Reconstitution of male germline cell specification from mouse embryonic stem cells using defined factors in vitro. Cell Death Differ..

[50] Nazm B.M., Ghasemi H., Narimanpour Z. (2020). An Efficient In Vitro Culture System to Amplify Spermatogonia Stem Cell Markers. Res. Mol. Med..

[51] Zheng Y., Tian X., Zhang Y., Qin J., An J., Zeng W. (2013). In vitro propagation of male germline stem cells from piglets. J. Assist. Reprod. Genet..

[52] Aponte P.M., Soda T., Teerds K.J., Mizrak S.C., Van de Kant H.J., de Rooij D.G. (2008). Propagation of bovine spermatogonial stem cells in vitro. Reproduction.

[53] Luo J., Megee S., Rathi R., Dobrinski I. (2006). Protein gene product 9.5 is a spermatogonia-specific marker in the pig testis: Application to enrichment and culture of porcine spermatogonia. Mol. Reprod. Dev..

[54] Silva J., Nichols J., Theunissen T.W., Guo G., Van Oosten A.L., Barrandon O., Smith A. (2009). Nanog is the gateway to the pluripotent ground state. Cell.

[55] Takahashi K., Yamanaka S. (2006). Induction of pluripotent stem cells from mouse embryonic and adult fibroblast cultures by defined factors. Cell.

[56] Arooj M., Rashid F.A., Gul A. (2013). Role of epigenetic modifications in stem cell regulatory regions (Oct4, Sox2 and Nanog) and cancer. IOSR J. Pharm. Biol. Sci..

[57] Nagano M., Ryu B.Y., Brinster C.J., Avarbock M.R., Brinster R.L. (2003). Maintenance of mouse male germ line stem cells in vitro. Biol. Reprod..

[58] Browne S., Jha A.K., Ameri K., Marcus S.G., Yeghiazarians Y., Healy K.E. (2018). TGF-β1/CD105 signaling controls vascular network formation within growth factor sequestering hyaluronic acid hydrogels. PLoS ONE.

[59] Niwa H., Miyazaki J.I., Smith A.G. (2000). Quantitative expression of Oct-3/4 defines differentiation, dedifferentiation or self-renewal of ES cells. Nat. Genet..

[60] Malaver-Ortega L.F., Sumer H., Liu J., Verma P.J. (2016). Inhibition of JAK-STAT ERK/MAPK and glycogen synthase kinase-3 induces a change in gene expression profile of bovine induced pluripotent stem cells. Stem Cells Int..

[61] Zhang Y.L., Li P.Z., Pang J., Wan Y.J., Zhang G.M., Fan Y.X., Wang F. (2019). Induction of goat bone marrow mesenchymal stem cells into putative male germ cells using mRNA for STRA8, BOULE and DAZL. Cytotechnology.

[62] Lee J.H., Engel W., Nayernia K. (2006). Stem cell protein Piwil2 modulates expression of murine spermatogonial stem cell expressed genes. Mol. Reprod. Dev..

[63] Saiti D., Lacham-Kaplan O. (2007). Mouse Germ Cell Development in-vivo and in-vitro. Biomarker Insights.

[64] Yuan L., Liu J.-G., Zhao J., Brundell E., Daneholt B., Hoog C. (2000). The murine SCP3 gene is required for synaptonemal complex assembly, chromosome synapsis, and male fertility. Mol. Cell.

[65] Anderson E.L., Baltus A.E., Roepers-Gajadien H.L., Hassold T.J., de Rooij D.G., van Pelt A.M., Page D.C. (2008). Stra8 and its inducer, retinoic acid, regulate meiotic initiation in both spermatogenesis and oogenesis in mice. Proc. Natl. Acad. Sci. USA.

